# Enhancing the genome editing toolbox: genome wide CRISPR arrayed libraries

**DOI:** 10.1038/s41598-017-01766-5

**Published:** 2017-05-22

**Authors:** Emmanouil Metzakopian, Alex Strong, Vivek Iyer, Alex Hodgkins, Konstantinos Tzelepis, Liliana Antunes, Mathias J Friedrich, Qiaohua Kang, Teresa Davidson, Jacob Lamberth, Christina Hoffmann, Gregory D. Davis, George S. Vassiliou, William C. Skarnes, Allan Bradley

**Affiliations:** 1 Wellcome Trust Sanger Institute, Wellcome Trust Genome Campus, Hinxton, Cambridge CB10 1SA UK; 2 MilliporeSigma St. Louis, Missouri, 2909 Laclede Ave, USA; 3 A Business of Merck KGaA, Darmstadt, 64293 Germany

## Abstract

CRISPR-Cas9 technology has accelerated biological research becoming routine for many laboratories. It is rapidly replacing conventional gene editing techniques and has high utility for both genome-wide and gene-focussed applications. Here we present the first individually cloned CRISPR-Cas9 genome wide arrayed sgRNA libraries covering 17,166 human and 20,430 mouse genes at a complexity of 34,332 sgRNAs for human and 40,860 sgRNAs for the mouse genome. For flexibility in generating stable cell lines the sgRNAs have been cloned in a lentivirus backbone containing *PiggyBac* transposase recognition elements together with fluorescent and drug selection markers. Over 95% of tested sgRNA induced specific DNA cleavage as measured by CEL-1 assays. Furthermore, sgRNA targeting GPI anchor protein pathway genes induced loss of function mutations in human and mouse cell lines measured by FLAER labelling. These arrayed libraries offer the prospect for performing screens on individual genes, combinations as well as larger gene sets. They also facilitate rapid deconvolution of signals from genome-wide screens. This set of vectors provide an organized comprehensive gene editing toolbox of considerable scientific value.

## Introduction

Bacterial and archaeal CRISPR (clustered regularly interspaced short palindromic repeats) have the capability of recognizing and specifically destroying invading viral DNA^1^. Following transcription of the CRISPR locus, the resulting RNA (crRNA) and trans-activating RNA (tracrRNA) hybridize into a crRNA-tracrRNA complex that together with a particular CRISPR-associated protein (e.g. Cas9) can bind and cleave foreign DNA^2, 3^. This elegant defence mechanism has been adapted for genome editing in higher eukaryotes^4^. The crRNA/tracrRNA have been fused to form a single guide RNA (sgRNA) with a “protospacer” sequence that can direct Cas9, a type II CRISPR endonuclease, in a sequence-specific manner to target DNA^5^. The specificity of the gRNA is enhanced by a protospacer-adjacent motif (PAM) on the opposite DNA strand while the gRNA binds the target strand by complementarity thereby guiding Cas9 to a specific nucleotide sequence where it generates a double-strand break (DSB). The break is subsequently processed by DNA repair mechanisms such as non-homologous end joining (NHEJ) or homologous recombination (HR)^6^. Template availability is a limiting factor for HR, thus NHEJ is the predominant repair pathway in mammalian cells^7^. After limited processing of the DNA ends, NHEJ promotes their joining resulting in error-prone repair with deletions or insertions (INDELs). In the context of coding exons and intron/exon boundaries, error prone repair is highly efficient at disrupting gene function^8, 9^.

The efficiency, specificity and adaptability of CRISPR/Cas9 have made it a particularly attractive tool for engineering genes and genomes of isolated cell lines, somatic tissues and the germ line of multiple species^10^. In contrast to the predecessor technologies which are directed to a specific nucleotide sequence through a protein-DNA interaction, the specificity of Cas9 is encoded in the short guide RNA. Consequently, it is relatively straightforward to generate multiple specificities in parallel by synthesizing and cloning pools of sgRNAs in a single experiment. Pooled libraries with 5 or more sgRNAs per gene have been used to conduct genetic screens in human and mouse genomes^4, 11^.

The major use of these pooled libraries has been in synthetic viability screens under conditions where there is a very strong selective phenotype^12^. In situations where the phenotype is more subtle, pooled libraries return hundreds or thousands of hits, requiring gene-by-gene analysis to distinguish true from false positives^13^. Synthetic lethality screens conducted with pooled libraries have been less successful because one is looking for depletion of a specific sgRNA. False positives occur when sgRNAs are underrepresented by chance while false negatives are generated by retention of sgRNAs which are due to the generation of heterozygous rather than homozygous mutants. Pooled libraries cannot be used where the screen relies on an assessment of cellular phenotype, such as the formation of protein complexes visualized as foci or tracking of fluorescently labelled proteins. Hence sgRNA libraries containing a defined set of guides in a clonal format for the entire mouse and human genomes constitute a valuable resource for conducting screens, as well as supporting verification of hits from pooled sgRNA screens^13, 14^.

Here we present the design and validation of genome wide arrayed sgRNA libraries for both the mouse and human genomes. Individual sgRNAs were cloned into a highly versatile backbone which supports delivery as a lentiviral particle or as a *piggyBac* (*PB*) transposon. These arrayed libraries cover 17,166 and 20,430 genes at a complexity of 34,332 and 40.860 sgRNAs for the human and mouse genome, respectively. These libraries have wide utility in many aspects of biology from whole genome screens to screen validation.

## Results

### Lenti-PB, a vector with dual delivery options

A construct was designed to facilitate stable integration into a destination genome via a lentivirus or as a *PB* DNA cut-and-paste transposon^15–17^. The lentiviral backbone provides the advantages of efficient single copy integration into a wide range of cell types and supports experimental designs in which complex pools of guide RNAs are used. The delivery of sgRNAs as a *PB* transposon from the same vector backbone is also highly efficient, determined by the transfection efficiency of the destination cell type^18^. The use of *PB* for delivery is methodically simpler and faster as only naked DNA is needed, avoiding the need to package, purify, titrate and deliver the vector through a lentiviral intermediate. Unlike lentiviral transduction, *PB* transposition usually results in multiple copy integration under most transfection conditions used^19^. Consequently, *PB* delivery results in much higher expression levels compared with those achieved with the same construct delivered by a lentivirus, facilitating sgRNA activity. Multiple copy integration is particularly useful when small numbers of different sgRNAs are co-delivered to the same cell, which is desirable for gene-interaction studies. If desired, cells with single copy insertions can also be isolated following *PB* delivery, although this is achieved at some loss of overall efficiency. The transposon can be seamlessly removed^19^ from such cells by re-expression of the *piggyBac* transposase (*PBase*), removing all selection and fluorescent markers and facilitating downstream use of the cell line.

**Figure 1:**
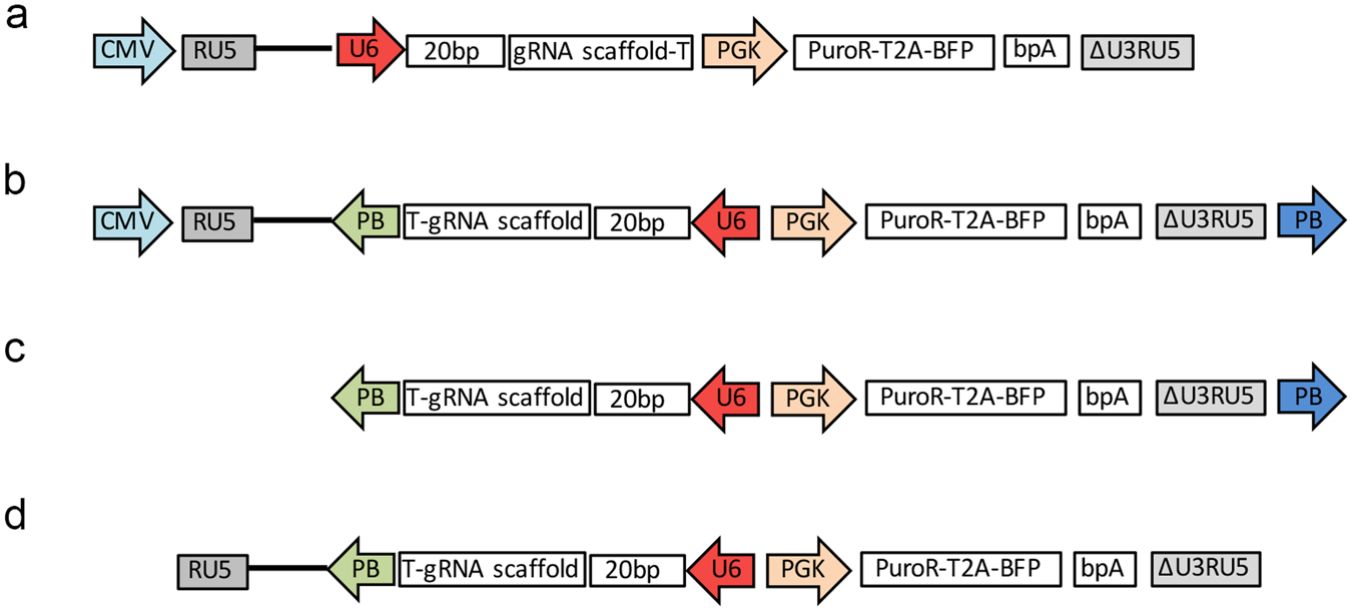
Lentiviral plasmid used in construction of the sgRNA library. (**a**) Schematic of original lentiviral construct pKLV-U6gRNA(*Bbs*I)-PGKpuro2ABFP described by Yusa *et al*.^11^. (**b**) Schematic of lentiviral construct Lenti-PB (pKLV-PB-U6gRNA(*Bbs*I)-PGKpuro2ABFP) used in the sgRNA library generation where the U6 promoter initiates sgRNA transcription on the opposite strand. PiggyBac LTRs were also added flanking the U6-gRNA-PGKpuro-2A-BFP cassette. (**c**) Schematic of PB transposon integrated U6-gRNA-PGKpuro-2A-BFP cassette. (**d**) Schematic of lentivirus integrated U6-gRNA-PGKpuro-2A-BFP cassette.

**Figure 2:**
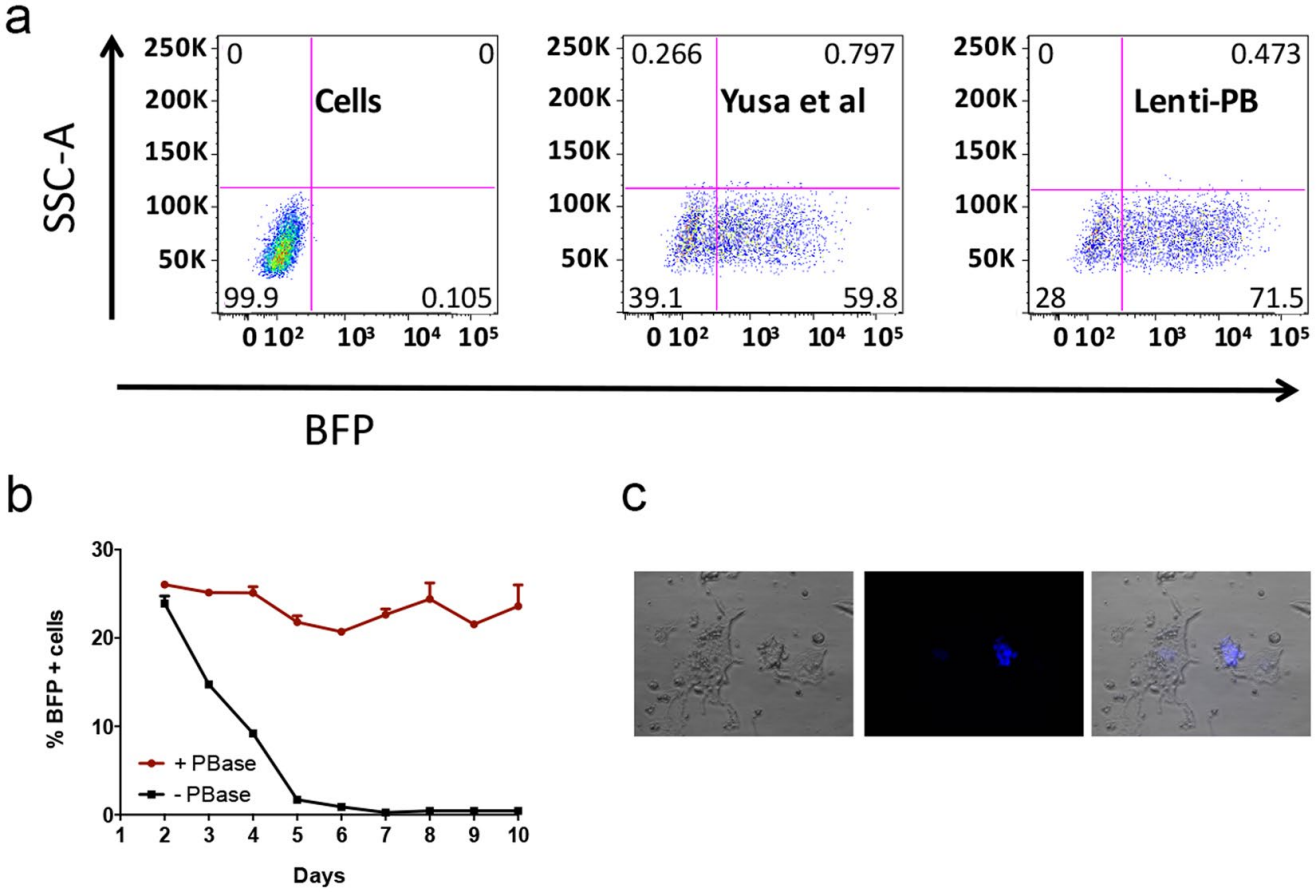
Lenti-PB stable expression by lentivirus or PB transposition. (**a**) BFP flow cytometry analysis of virus packaging and production of Lenti-PB construct compared to the original lentiviral construct in mouse ES cells. No puromycin selection was added to these cells. (**b**) Graph indicating percent of BFP positive cells over ten-day time points of Lenti-PB plasmid co-transfected with PB transposase expressing plasmid (red) for stable integration or with p-bluescript plasmid (black) for transient expression. (**c**) Example of BFP positive mouse embryonic stem cells transfected with Lenti-PB.

The lentiviral backbone used by *Koike-Yusa H. et al*.^11^ was modified as described (Fig. 1a and b). To minimize the impact of transcription by the U6 promoter driving sgRNA expression on lentivirus production, the U6 promoter was cloned in reverse orientation with respect to the CMV promoter which drives the mature lentiviral transcript (Fig. 1b). One *PB* transposon inverted terminal repeat (ITR) was inserted upstream of the U6-gRNA-Pgk-Puro-T2A-BFP cassette, while the second was positioned downstream of the lentiviral 3′ LTR (Fig. 1b)^19^. To evaluate the effect of the *piggyBac* sequence on virus titre we compared the lentivirus-PB construct with the original vector using mouse embryonic stem cells (mESC). Flow cytometry analyses of blue fluorescent protein (BFP) positive mESCs illustrate no difference in viral titers produced using either construct (Fig. 2a). As configured the inserted provirus carries a single PB 5′ ITR (Fig. 1d), which is not a substrate for *PBase*. However, the plasmid vector carries both *PB* ITRs, thus in the presence of *PBase* the sequences flanked by the *PB* ITRs are efficiently transposed from the construct and stably integrated into the desired genome^20^ (Fig. 2b and c).

### gRNA library design and synthesis

**Figure 3:**
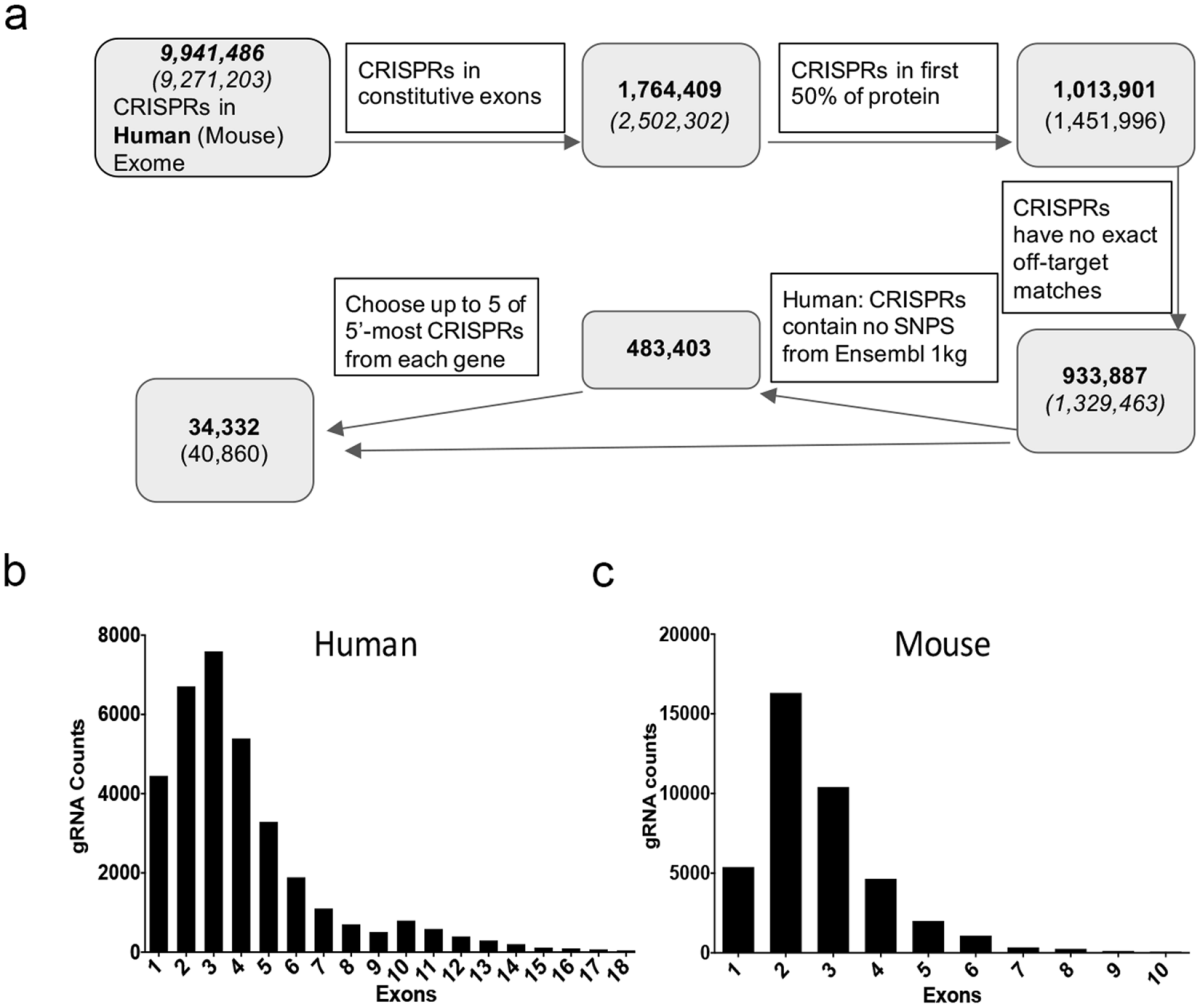
Human and mouse sgRNA arrayed library design. (**a**) Schematic illustrating sgRNA genome wide library design work flow. (**b**,**c**) Graphs illustrating the number of sgRNA designed per gene exon in the human and mouse sgRNA arrayed libraries.

For the human and mouse genomes two sgRNAs were designed to target each protein coding gene. For the human library, 34,332 sgRNAs targeting 17,166 protein coding genes were designed according to the following rules (Fig. 3a, Table S1): each protein coding gene in Ensembl v 75 *Homo sapiens* gene built (GRCh38) was inspected to find and rank its constitutive coding exons – those exons shared by all coding transcripts of a gene (1,764,409). Within these exons, we identified all CRISPR sites and restricted the design to the first 50% of the protein-coding sequence, (1,012,436). Each site was scored for possible off-targets with up to 4 mismatches^21^. Any sgRNA with an exact match anywhere else in the genome was removed (933,281). In order to minimize the effect of different genetic backgrounds on the sgRNA function, we discarded any CRISPR site containing a single nucleotide polymorphism found in the Ensembl Thousand Genome Phase 1 variant set (450,484 discarded). Finally, two of the 5′-most sgRNA’s were chosen for each gene. Evaluation of the exons targeted in the whole library illustrates that over 85% of the sgRNA target up to exon 5 of any gene with the largest number of sgRNAs targeting exon 2 and 3 (Fig. 3b).

The mouse library was similarly designed with 40,860 sgRNA targeting 20,430 genes (Fig. 3a, Table S2). Protein coding genes in the Ensembl v72 *Mus musculus* gene build (GRCm38) were screened and inspected for their constitutive coding exons. Over 78% of the designed sgRNA target exons 1–3 of the respective genes (Fig. 3c). sgRNAs with an exact match anywhere else in the genome were removed, leaving 1,329,463 potential sgRNA designs. Since most mouse genomes used in research are either C57BL/6J or have been sufficiently annotated for genomic variation, SNP interference for downstream experiments can be predicted or avoided^22^. We therefore decided to not filter for SNPs for the mouse genome gRNA designs as it would significantly reduce the total pool.

**Figure 4:**
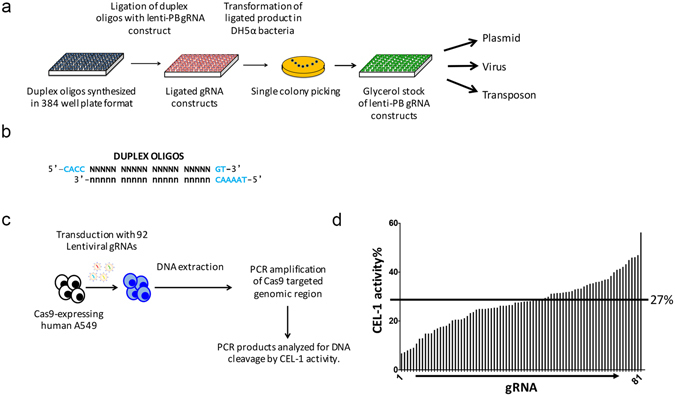
(**a**) Schematic showing the cloning strategy of the sgRNA libraries in multi well plate format. (**b**) Duplex oligos synthesized for cloning in the lentiviral-PB sgRNA vector. (**c**) Schematic illustrating the experimental strategy for validation of 92 sgRNAs chosen from the human genome wide sgRNA library. (**d**) CEL-1 activity results indicating the cutting efficiency of 81 out of 85 samples which produced a PCR product indicating success rate of 95% functional sgRNAs. Average cutting efficiency of 27% is observed.

Traditional restriction ligation cloning was used to assemble both the human and mouse libraries in a 384 well plate format (Fig. 4a,b). Quality of the individual sgRNA vector coordinates, as well as performance of the vector and gene editing efficiency was assessed. Ninety-two sgRNA vectors which target 46 different human genes were selected at random. Each vector was sequenced which confirmed their identity was correct for each clone examined and fidelity of the oligonucleotide synthesis was 100% correct. Each clone was individually packaged as a lentivirus and used to infect human A549 cells expressing Cas9 (Fig. 4c). Of the 92 different sgRNA vectors packaged, all successfully produced infective lentivirus with titres greater than 10^6^/ml assessed by BFP expression in flow cytometry. Without optimisation average transduction efficiencies of 41% were achieved (range 22% to 55%).

To assess gene editing efficiency BFP-positive cells were selected with puromycin, sgRNA target sites were amplified and assessed by CEL-1 cleavage^23^. A total of 85 amplicons were generated with first pass primer designs (Table S3) and of these 81 (95%) gave definitive CEL-I cleavage from lentiviral transduced BFP-positive populations (Table S4). The Cas9 cutting efficiency from clones with lentivirally delivered vectors ranged from 7% to 56% with an average of 27% (Fig. 4d, Table S4).

### Phenotypic library validation with GPI anchor protein pathway genes

To further validate the genome editing performance of the arrayed library we selected 7 human genes (14 sgRNA’s) and 6 mouse genes (12 sgRNA’s) that are components of the glycosylphosphatidylinositol (GPI)-anchor synthesis pathway^24, 25^. Loss of function of these genes results in loss of GPI-anchored proteins on the cell surface. The bacterial Aerolysin toxin which binds specifically to GPI structures was conjugated with Alexa 488 fluorochrome (FLAER) which labels cells with GPI anchored proteins. Cells which lack GPI anchor proteins are FLAER-negative and these two populations can be efficiently quantified by flow cytometry^26^.

**Figure 5:**
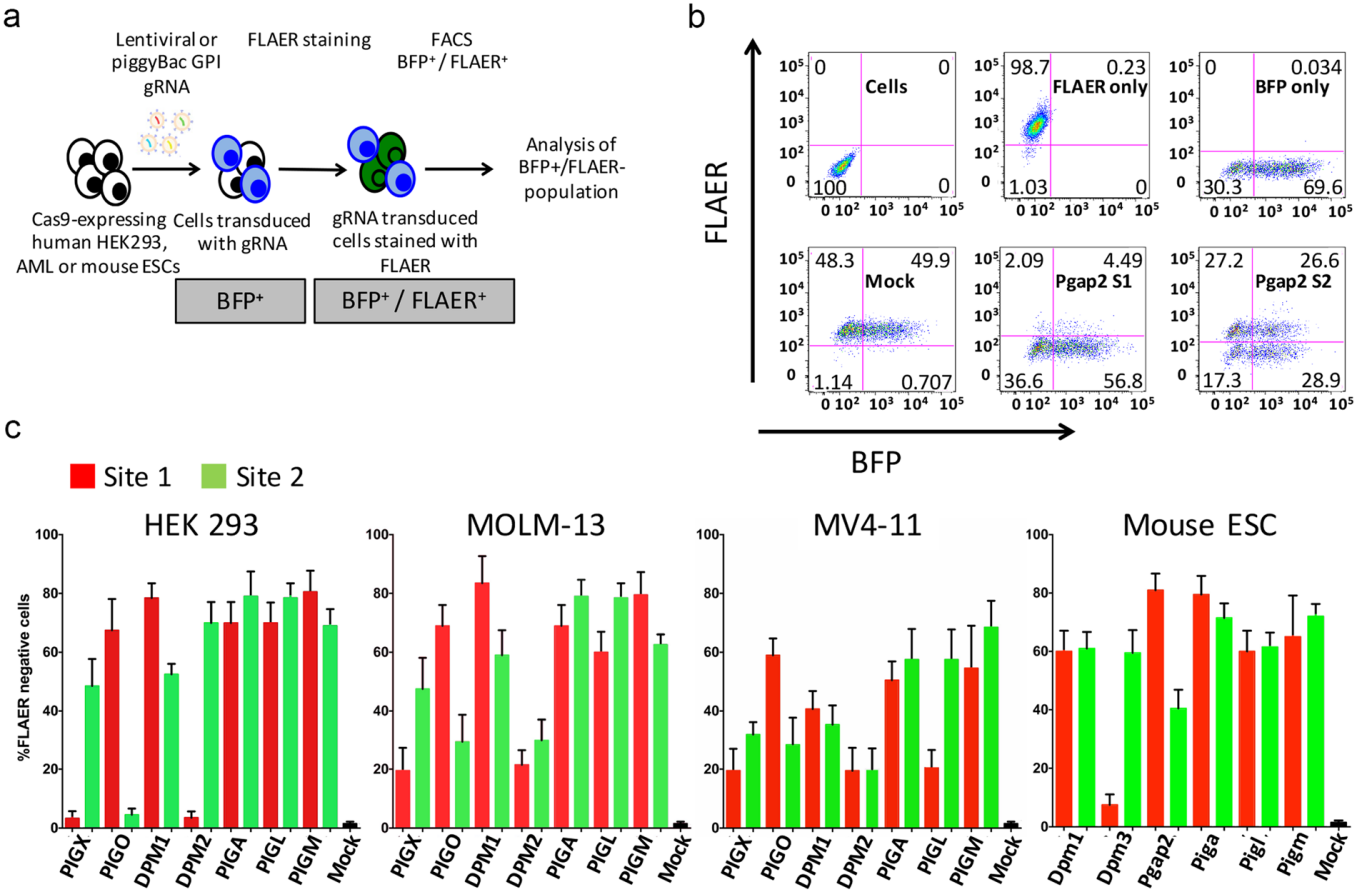
(**a**) Schematic illustrating the experimental design for the GPI anchor protein pathway sgRNA phenotypic evaluation. (**b**) Example of flow cytometry analysis of FLAER stained cells using mouse ES cells transfected with sgRNAs against Pgap2 gene targeting site 1 and site 2. The sgRNA expressing cells were selected with puromycin from 24 hours post transfection and selection was maintained for 2 days. Cells were analysed 6 days post transfection. Mock experiment was performed with non-targeting gRNA. Cells: untransfected and unstained cells. FLAER only: untransfected and FLAER stained cells. BFP only: transfected cells with a non targeting sgRNA construct and unstained. Mock: transfected cells with a non targeting sgRNA construct and FLAER stained. Pgap2 S1 and S2: transfected cells with sgRNAs targeting site 1 and site 2 of the Pgap2 gene and FLAER stained. (**c**) Flow cytometry results for BFP positive/FLAER negative human HEK 293, MOLM-13, MV4-11, and mouse embryonic stem cells. *PB* transposition of sgRNA constructs was used for human HEK293 and mouse embryonic stem cells, lentiviral transduction of sgRNA constructs was used for the acute myeloid leukaemia (AML) cell lines, MOLM-13 and MV4-11.

The human sgRNA vectors were stably integrated in the genomes of three different Cas9 expressing cell lines. *PB* transposition was used for HEK293 cells, lentiviral transduction was used for the acute myeloid leukaemia (AML) cell lines, MOLM-13 and MV4-11^27, 28^ while mouse sgRNA vectors were introduced into the genomes of Cas9-expressing ES cells by *PB* transposition (Fig. 5a). Two day puromycin selection was initiated 24 and 48 hours post *PB* transfection and viral transduction, respectively. Flow cytometry of FLAER stained HEK293 and mouse ES cells revealed all the selected genes were efficiently edited with at least one of the selected sgRNAs (Fig. 5b,c). All of the sgRNAs used in the AML cell lines were functional and demonstrated successful gene editing. The gene editing efficiencies achieved in these experiments were very high, greater than 50% for most of the sgRNAs tested in all cell lines except MV4-11 (Fig. 5c).

In Fig. 5b, loss of FLAER staining indicates successful editing of the Pgap2 gene by two different sgRNAs as compared to an unrelated (mock) sgRNA (bottom panel, 93.4% and 46.2% for sgRNAs S1 and S2, respectively). However, a significant subset of the FLAER negative, edited cells shows no BFP expression (bottom left quadrant), suggesting the seemingly conflicting result of gene editing without sgRNA expression. This is likely due to the subsequent loss of the sgRNA expression plasmid after the initial transfection: A brief period of sgRNA expression is enough to induce gene editing; however, the relatively short duration of puromycin selection (2 days) combined with the re-integration efficiency of *PBase* of around 40%^29^ allows for transient-only transfection in a subset of cells, which then appear sgRNA-negative, but nevertheless edited. Prolonging antibiotic selection almost completely eliminates these cells, which over time – together with BFP expression – also lose their resistance to puromycin, as shown in Supplementary Figure 1 where selection was maintained for 7 days in an otherwise identical experiment.

## Discussion

We here present the first human and mouse genome wide CRISPR-Cas9 sgRNA libraries for arrayed screening. The libraries comprise 34,332 and 40,860 unique sequence-validated sgRNAs targeting coding genes in the human and mouse genomes, respectively. The libraries are arrayed in 96 micro-well plates and in contrast to pooled libraries, these clonal libraries make it possible to study complex phenotypes in parallel, such as the subcellular localization of a fluorescent reporter or the dynamics of drug sensitivity, by applying the library to cells grown in an arrayed format using automated equipment.

Efficient delivery and genome integration of the sgRNA expression cassette can be achieved using the constructs from these arrayed libraries through lentivirus transduction or *PB* transposition. By coupling sgRNA expression to that of the BFP reporter gene, transfected or transduced cells can be directly visualized and/or sorted to identify cells that are potentially editable. However, editing is not 100% efficient, so methods are required to identify within a population of BFP sorted cells those in which all alleles have been edited to a complete gene loss-of-function status.

The sgRNAs in the library were designed to mediate disruptive loss-of-function mutations in coding genes and to take account of sequence variation in the human (but not the mouse) genome. Pooled sgRNA libraries typically contain 5 or even more sgRNAs per gene, in part to compensate for sgRNAs that fail to induce gene editing, but mainly because the distribution of sgRNA representation in the pool requires more sgRNAs per gene to allow robust statistical data analysis. Although the arrayed sgRNA libraries described herein contain two sgRNAs per gene, the expected rate of non-editing is on the order of 50 genes per full library (assuming a rate of 5% non-functional sgRNAs), with the great advantage of being able to update the libraries by replacing those sgRNAs based on experimental feedback. Furthermore, sampling of clones in these libraries targeting human and mouse genes involved in the GPI anchor protein pathway induced loss of FLAER staining, indicating loss of gene function in a large proportion of cases, although some guides did exhibit different activities between human cell lines. In most cases the sgRNA was effective in at least one cell line tested, confirming the sgRNA design is correct. However, loci will vary in their chromatin conformation state between cell lines, impacting access of the Cas9-complex in a cell line-specific manner^30^. Nucleotide sequence variation in the sgRNA target site as well as ploidy are also likely to contribute to differences in editing efficiency between cell lines^31^.

All screens with CRISPR-Cas9 described to date have employed a forward genetics approach, where “dominant” phenotypes are directly selected and/or enriched, and the causative genetic modifications revealed by overrepresentation in the selected pool. Such screens invariably produce a long list of candidate genes which need to be further validated individually. The clonal library described here will greatly facilitate this process. When the candidate list is extensive, sub-libraries can conveniently be selected from the master library and either be used individually or as a pool. Cross - species validation is also enabled from human to mouse or vice-versa by the availability of a complete clone set for both species. The application of arrayed CRISPR libraries in reverse genetic loss-of-function screens in multi-well plates will significantly expand the breadth of phenotypic screens that can be conducted genome - wide.

## Methods

### Selection of CRISPR sites for Mouse

Each protein-coding gene in the Ensembl v.72 *Mus musculus* gene build (GRCm38) was inspected to find the most 5′ constitutive coding exons (exons included in all coding transcripts). All possible CRISPR (N20NGG) sites within these exons were selected and re-aligned to the mouse genome using the exonerate^32^ aligner with a sensitive matching criterion (minimum align score = 80), to find potential off-target sites in the genome which were identical in the ‘seed’ sequence - the 12 bp adjacent the PAM. To allow for variation in the first bp of the PAM (NGG), four separate alignments were performed. Each possible off-target site was classified by its location (exonic, intronic or intergenic). Finally, we selected the two CRISPR sites inside each chosen exon that had the minimal number of potential exonic off-targets.

### Selection of CRISPR sites for Human

Each protein coding gene in Ensembl v 75 *Homo sapiens* gene build (GRCh38) was inspected to find and rank its constitutive coding exons. Within these exons, we identified all CRISPR sites (N20NGG), restricting to those within the first 50% of the protein sequence. Each site was scored for possible off-targets using the CRISPR-Analyser package in the WGE database^21^. This package uses an exhaustive search method, allowing for up to four mismatches anywhere in the CRISPR’s sgRNA sequence (N20). Any CRISPR with an exact off-target match in the genome was discarded, as was any CRISPR site containing a SNP found in the Ensembl Thousand Genome Phase1 variant set. The remaining CRISPRs were ranked within the gene from 5′ to 3′ and the first two CRISPR sites chosen. For any genes lacking two CRISPRs chosen this way, we re-selected from all possible CRISPRs in the constitutive coding exons but allowing for SNPs from the Thousand Genomes variants.

### Cell culture and transfection

Male JM8 mouse ES cells^33^ were cultured at 37 °C with 5% CO_2_ in M15: Knockout DMEM supplemented with 15% fetal calf serum (FCS), 2 mML-glutamine, 50 U/ml penicillin, 40 μg/ml streptomycin, 100 μM beta-mercaptoethanol, and 1,000 U/ml of recombinant mouse leukemia inhibitory factor (LIF;Millipore). Cell media were changed daily unless other-wise specified. ES cells were cultured on a layer of gamma-irradiated (60 Gray) SNL76/7 STO feeder fibroblasts^34^. 293FT (Invitrogen) were cultured in DMEM containing 10% FBS and 1% GlutaMax. AML cell lines MOLM-13, and MV4-11 were grown in RPMI 1640 with 10% FBS. Transfection of mouse ESCs and human HEK 293 cells was carried out using Lipofectamine LTX (Invitrogen) according to the manufacturer’s instruction. Briefly, 100 ng of plasmid DNA (90 ng *PB* sgRNA construct and 10 ng *PBase* construct) and 0.1 μl of the PLUS reagent were mixed into 10 μl OPTI-MEM (Invitrogen) and incubated for 5 min at room temperature. 0.3 μl of the LTX reagent was diluted into 10 μl of OPTI-MEM and combined with the DNA:PLUS mixture. This was incubated for 30 min at room temperature. Subsequently, 15,000 ESCs or HEK 293 cells suspended in 80 μl OPTI-MEM were mixed with 20 μl of the DNA:PLUS:LTX mixture and plated onto a well of a 96-well plate. These cells were incubated for 1 hour at 37 °C. The transfection mixture was then removed and 150 μl of medium were added. The transfected cells were cultured for 6–7 days before relevant functional analysis. Transfected cells were selected with puromycin 24 hours post transfection for either 2 days or throughout the cell culture term. The non-targeting sgRNA control used in experiments involving mouse cells is “GCTATATACTCGACACCCAA” and the non-targeting sgRNA control used in experiments involving human cells is “ATTTTCGTACCCTGGGACGC”.

### Flow cytometry

Fluorophore (Alexa488)-labelled aerolysin (FLAER) was purchased (VH bio) and used for cell staining at 25 nM in 1% BSA in PBS for 20 min at room temperature. The stained cells were analysed on the LSRII or LSRFortessa instrument (BD) and Beckman Coulter CytoFLEX. Data were subsequently analysed using FlowJo.

### Lentivirus production and transduction

Three μg of a lentiviral vector, 9 μg of ViraPower Lentiviral Packaging Mix (Invitrogen) and 12 μl of the PLUS reagent were added to 3 ml of OPTI-MEM and incubated for 5 min at room temperature. Thirty-six μL of the LTX reagent were then added to this mixture and further incubated for 30 min at room temperature. The transfection complex was added to 80% confluent 293FT cells and incubated for 3 hours. The medium was replaced with fresh medium at 24 h post transfection. Viral supernatant was harvested at 48 h post transfection and stored at −80 °C. Transduction of ESCs was performed in suspension as follows: 15,000 ESCs and diluted virus were mixed in 100 μl of the ESC medium containing 8 μg ml-1 polybrene (Millipore), incubated for 30 min at 37 °C in a well of a round-bottomed 96-well plate, plated onto a well of a gelatin coated 96-well plate and cultured until analyses.

Transduction of AML cells was performed in suspension as follows: 15,000 AML cells and diluted virus were mixed in 100 μl of RPMI 1640 with 10% FBS and 8 μg ml-1 polybrene (Millipore), incubated for 30 min at 37 °C in a well of a round-bottom 96-well plate, plated onto a well of a 96-well plate and cultured until functional analyses 12 days post viral transduction. AML cells containing the sgRNA construct were selected with puromycin 48 hours post transduction and selection was maintained for two days.

### Genome-wide mouse and human sgRNA lentiviral-*PiggyBac* library construction

Top and bottom 26-nt oligonucleotides (Supplementary Tables 1 and 2) were individually mixed at 5 μM each in 10 mM Tris-HCl (pH8.0) and 5 mM MgCl_2_ in a total volume of 50 μl in 384 well plates. The mixture was incubated at 95 °C for 5 min and cooled to room temperature. The duplex oligonucleotides were then cloned into the *Bbs*I site of pKLV-PB-U6gRNA(*Bbs*I)-PGKpuro2ABFP (Lenti-PB) in 384 well plate format.

Cloned plasmids were then transformed into Dh5α bacteria, and then streaked onto agar plates and single colonies were picked. All clones were sequence verified after picking the single colony. This step eliminated any empty vector contamination. The glycerol stock was created from the growth of the single colonies. In addition, for any clone that did not pass sequencing quality control we repeated the process to regain all clones, in order to achieve 100% sequence verification of the entire library.

All high throughput liquid handling was performed using the automated 96 and 384 channel pipettor Felix (Cybio).

### Genome-wide mouse and human sgRNA lentiviral-*PiggyBac* library distribution

Distribution of the human and mouse CRISPR arrayed libraries is performed by MilliporeSigma (a business of Merck KGaA, Darmstadt Germany). Complete libraries are referenced on www.sigmaaldrich.com as product numbers HSANGERG and MSANGERG for the glycerol stocks and HSANGERV and MSANGERV for the viral particles, respectively. Individual gRNA or mutli-gRNA subsets are also available for distribution.

## Electronic supplementary material

Supplementary Figure 1

Supplementary Table 1

Supplementary Table 2

Supplementary Table 3

Supplementary Table 4
