# Supplementary material for: Enhancing the genome editing toolbox: genome wide CRISPR arrayed libraries
Source: Sci Rep. 2017 May 22;7:2244. doi: 10.1038/s41598-017-01766-5 (PMC5440395; doi:10.1038/s41598-017-01766-5)
Supplement: Supplementary file 1 — Supplementary Figure 1 [file 41598_2017_1766_MOESM1_ESM.pdf]

Enhancing the genome editing toolbox: genome wide CRISPR arrayed libraries

Emmanouil Metzakopian[1], Alex Strong[1], Vivek Iyer[1], Alex Hodgkins[1], Konstantinos Tzelepis[1], Liliana Antunes[1], Mathias Friedrich[1], Qiaohua Kang[2], Teresa Davidson[2], Jacob Lamberth[2], Christina Hoffmann[2], Gregory D. Davis[2], George S. Vassiliou[1], William C. Skarnes[1], Allan Bradley[1]*.

1. Wellcome Trust Sanger Institute, Wellcome Trust Genome Campus, Hinxton, Cambridge CB10 1SA, UK.
2. MilliporeSigma, St. Louis, Missouri, 2909 Laclede Ave

* Correspondence should be addressed to: A.B (abradley@sanger.ac.uk)

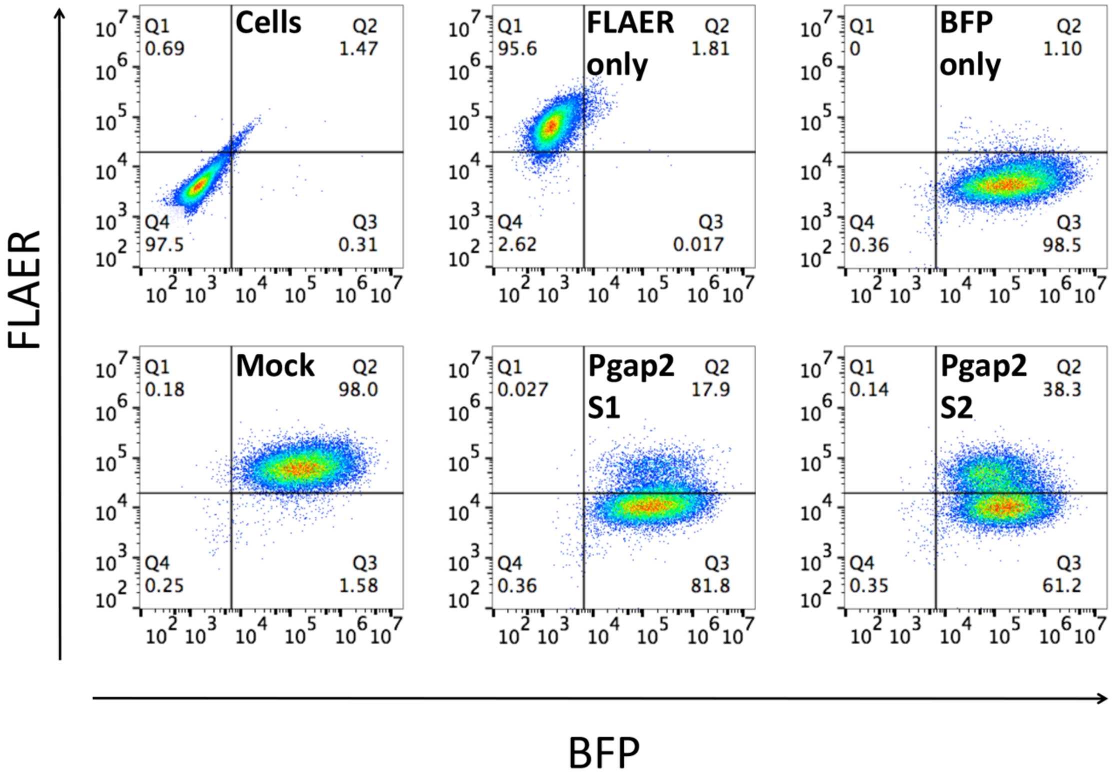
FLAER
BFP
Cells
Q1 0.69
Q2 1.47
Q4 97.5
Q3 0.31
FLAER only
Q1 95.6
Q2 1.81
Q4 2.62
Q3 0.017
BFP only
Q1 0
Q2 1.10
Q4 0.36
Q3 98.5
Mock
Q1 0.18
Q2 98.0
Q4 0.25
Q3 1.58
Pgap2 S1
Q1 0.027
Q2 17.9
Q4 0.36
Q3 81.8
Pgap2 S2
Q1 0.14
Q2 38.3
Q4 0.35
Q3 61.2

Supplementary Figure 1. Flow cytometry analysis of FLAER stained cells using mouse ES cells transfected with sgRNAs against Pgap2 gene targeting site 1 and site 2. The sgRNA expressing cells were selected with puromycin from 24 hours post transfection throughout the cell culture term. Cells were analysed 7 days post transfection. Mock experiment was performed with non-targeting sgRNA. Cells: untransfected and unstained cells. FLAER only: untransfected and FLAER stained cells. BFP only: transfected cells with a non-targeting sgRNA construct and unstained. Mock: transfected cells with a non targeting sgRNA construct and FLAER stained. Pgap2 S1 and S2: transfected cells with sgRNAs targeting site 1 and site 2 of the Pgap2 gene and FLAER stained.
